# Diagnosis and Treatment of Pott’s Disease of the Spine

**Published:** 1866-08

**Authors:** F. O. Earle

**Affiliations:** Chicago, Ill.


*Diagnosis and Treatment of Pott's Disease of the Spine.* By F. O. EARLE, M. D., Chicago, Ill.

It has seemed to me somewhat remarkable that the well-defined symptoms of this disorder, which is so common among all classes of society, so disastrous in its results when neglected, but so amenable to treatment in its earlier stages, should be almost entirely overlooked, as such, by the general practitioner in this age of our profession, when diagnosis is made so prominent a part of a thorough medical education. And for this reason, I have been induced to prepare this paper, hoping thereby to attract the attention of practitioners more forcibly to the importance of an early recognition of this terrible malady, and to urge upon them the necessity for its prompt, thorough and effectual treatment by proper mechanical means.

What, then, are the symptoms by the presence of which we are to be guided to a correct diagnosis in these cases, where an accurate decision is of such vital importance to the future welfare of the patient?

First. Pain,—*not*, as is generally supposed, in the back or along the spine—which is very rarely the seat of tenderness, unless caused by the manipulations of the physician in his vain endeavors to find a sore spot,—but on the anterior portion of the trunk, and generally at the epigastrium, though sometimes at the hypogastrium, and occasionally in the thoracic region, near the costo-sternal articulations, or at the borders of the ribs, being modified somewhat by the point at which the disease is situated. So constant is this symptom, that it may be considered pathognomonic of the disease under contemplation. The pain is rather paroxysmal in character, and is intense and agonizing, often causing the most piercing cries to escape the lips of the sufferer. In some cases it seems to be excited by slight causes, while in other instances it comes on spontaneously. It is sometimes present in the evening, but in other cases is complained of in the morning on rising from bed, or it may only be noticed when the patient is fatigued.

This prominent symptom of a vital disorder is very often mistaken for the whole disease, and the patient is accordingly treated for worms or inflammation of the bowels, gastritis, or indigestion, until the attention of the medical adviser is fully aroused to the serious nature of the malady which he has been vainly endeavoring to combat with inert remedies, by the actual appearance and rapid increase of the hideous deformity which, sooner or later, results from ulcerative inflammation of the spine.

Second. A peculiar and characteristic attitude and gait, which is adopted for the purpose of avoiding as much as possible all shocks and concussion to the spine. The head is thrown backward as well as the shoulders, which are also slightly elevated; the elbows, hips and knees are flexed, and the toes are generally turned in. The patient steps very carefully, often resting one hand (generally the left) upon the knee, and when standing, will lean upon a chair or table, or his mother's lap, for support. If asked to pick up some object from the floor, he will squat down by the side instead of in front.

In connection with these more prominent signs, we often find great irritability of temper and nervous excitability, appetite extremely capricious or almost entirely lost, tumefaction of the abdomen, general debility and indisposition, and the gradual development of a cachectic condition.

If the disease be situated in the dorsal region, the respiration will be short and labored.

If the physician should find one or more of the symptoms which have been enumerated present, but should still be in doubt as to the true nature of the affection, let him view the spinal column from the side, and he will notice a straightening of the spine at some point instead of the normal curve; or, if the disease is a little farther advanced, a bulging outward, with straight lines above and below, converging towards the point where the disease is situated. If he fails in detecting either of these appearances, let him take a full view of the back, and if there is a slight deviation of the spine to one side without any true course, but in its place an angle, and the line of spinous processes above and below converging to that angle as to a focus, he may be sure that mischief is going on at that point.

This lateral *deviation* is likely to be confounded with true lateral *curvature* of the spine, but there is one sure method of settling this doubt, if it should arise in the mind of the physician. If disease of the spine exists, there is generally, in connection with the *apparent* curvature, a very slight projection backward of one spinous process, and by grasping the patient's hips firmly and directing him to stretch the arms straight above the head, and carry the head and trunk forward without flexing the spine, the lateral deviation will disappear, but the projection will still remain visible, showing beyond the possibility of a doubt that the vertebræ at this point are diseased.

But the physician should not wait for the manifestation of these physical signs. It is a well known and generally recognized law—illustrations of which often occur in the practice of every physician—that lesions of nervous centres and main trunks exhibit their earliest manifestations at a distance from the seat of irritation, in the extremities of the nerves which have their origin at this point.

A persistent, severe, paroxysmal pain in the region of the stomach, which resists all ordinary and well known remedies, should immediately attract the attention of the intelligent practitioner, who has a knowledge of the existence of the above-mentioned law, to the spinal column as the focus of this abnormal sensation.

Having satisfied ourselves that progressive ulcerative inflammation of the bodies of the vertebræ or of the intervertebral substance exists, what are the indications for treatment?

Evidently, first, that which has of late years been so successfully adopted in the treatment of that similar affection, morbus coxarius, namely: to relieve the pressure at the point of disease; and, secondly, to sustain the general tone and vigor of the patient's system, by the adoption of such hygienic measures and the administration of such remedies as the nature of the case seems to require.

The last named indication is second not only in order but in importance, for it is a fact founded upon experience, that, if the first indication is promptly and effectually fulfilled, the accomplishment of the second follows as a natural result in the majority of cases, without any attempt to combat the accompanying constitutional symptoms by general medication.

How, then, may the first, and by far the most important, requirement in these cases be properly answered?

By the introduction of setons and issues, or the application of moxæ and the actual cautery? I cannot deny that some kind of counter-irritation is useful in certain forms of disease, but I do positively maintain that no possible benefit can be derived from the adoption of such measures in the affection under consideration.

Without entering at the present time into an examination of the various theories, which have been held by pathologists of our own and former periods, with regard to the causation of this malady, we must accept the fact that we have a morbid process going on, which is gradually but surely undermining the constitution; disturbing rest and appetite; causing hectic, and producing a hideous deformity. Now, is it reasonable to suppose that we shall be able to control this unhealthy action, with all its accompanying horrors, by the institution of any form of treatment which will cause a still further drain upon the system, which is already taxed almost beyond endurance? It seems to us that mature reflection will disclose the utter uselessness of such measures.

In most surgical works, the recumbent posture, either in the prone or supine position, has been very strongly insisted upon as absolutely necessary in this disease. Neither of the indications are properly fulfilled by this method of treatment, and therefore it is objectionable, except under one condition which is liable to occur, and which will be hereafter mentioned. The prone position does not remove the superincumbent weight from the bodies of the diseased vertebræ, and consequently the deformity is increased rather than diminished; the weight of the trunk is supported on the thorax and abdomen; the play of the ribs, and of the abdominal and thoracic muscles and viscera, is restricted; the thorax becomes flattened, and the digestive, respiratory, and circulatory functions necessarily impeded. This system, with all its lingering horrors, is now happily abandoned, though confinement to the bed in the supine position, which is only less harmful, is still recommended and adopted by many surgeons, as being the only practicable means of relieving the diseased surfaces from the weight and pressure to which they are constantly subjected, if the body is held erect. But, while the tendency is to produce this effect, it is done at the expense of the general health of the patient, as it materially interferes with those various hygienic measures which are so necessary to subjects of this class. Besides this, the functions of the skin are impeded by the constant pressure to which it is exposed, and organic lesions of the soft parts are induced, while the natural action of the muscles is curtailed, and those organs consequently become weak and atrophied.

Many mechanical arrangements have been devised for the relief of this class of patients, but they have generally been constructed on the same principle, and that a false one, not recognized in mechanics, so that we need not wonder at the want of success which has attended their use. Nearly every instrument coming under my observation, has been made with the one idea of producing counter-extension from the hips to the shoulders, by means of crutches passing from a hip-band and resting under the arm-pits.

There are two insurmountable obstacles in the way of producing any beneficial result by this means:

First, the hips are never broad and full enough to afford a basis of support, and

Second, we can find no fixed point above.

Any force applied under the arm-pits only raises the weight of the arms themselves, while it seriously interferes with the circulation in those extremities, which, in connection with the pressure downward upon the hips, becomes in a short time intolerable.

An apparatus, to be thoroughly effective in cases of this kind, should admit of such freedom of action on the part of the patient as will conduce to his comfort and welfare, while, at the same time, it absolutely protects the diseased portion of the spine from pressure and the shocks to which it is constantly liable.

Such an instrument has been invented by Dr. Charles F.

Taylor, of New York city, and used by him and others with very marked success in the treatment of this disease during the past few years.

This very useful surgical appliance consists of a well-padded hip-band, which passes rather more than half-way around the trunk, midway between the great trochanters and crista ilii. From this, two narrow steel pieces pass up the back on either side of the spine, which are fastened at the top to a double-T-shaped piece of steel, of the same width and thickness as the uprights, to each cross of which is attached a strap which passes over the shoulder and under the arm. The shape of the cross-piece prevents the ligaturing of the arms, which would not only be painful but injurious. Opposite the point of disease are two broad steel plates, which are fastened above and below to the two uprights by means of stop-hinges, which absolutely prevent motion *forward*, but allow the most unrestrained use of the muscles in the *backward* direction, thus bringing the erector-spinæ into action, and so causing their development, and making them a means of sustaining a portion of the weight of the body, and thus assisting in a manner to overcome the curvature. I have seen this effect produced in many cases. Upon these plates are fastened the pads, which press not directly upon the spine but upon either side of it. These are made of canton flannel, and filled with wool or ground cork. The latter is best, as it is quite as soft as wool, and is not so liable to pack and become saturated with perspiration. The shoulder-straps and the strap which passes from either end of the hip-band, should be made of firm—*not* elastic—webbing, and provided with pads to protect the parts from abrasion, and all these should be renewed often to insure cleanliness and efficiency. The strap, which is attached by means of buckles to either end of the hip-band, passes below the abdomen, and to it is sewed a broad bellyband, which passes up in front, and is buckled *loosely* to the uprights by each upper corner. This proves grateful to the patient, as it sustains the pendulous abdomen, and does not interfere with the respiratory, circulatory or digestive functions. Passing through the uprights, above and below the hinges, are little screws which play upon the pad plate, forcing open the hinges, thus increasing the action of the instrument when desirable.

It is not claimed for this apparatus, neither can it be for any other, that the mere application of it is to do anything towards the cure of so formidable a malady. Any surgical appliance should not only be constructed upon correct mechanical principles, but it is also necessary that it should be carefully fitted to each case, which should be constantly watched and attended, that any indications for treatment which may arise may be promptly answered by corresponding changes in the form and relative bearings of the instrument. And these necessary modifications should be made, in all cases, by the physician or surgeon himself, who is the only proper person to administer any medical or surgical treatment.

The apparatus described above may be modified to suit the requirements of any case, in whatever portion of the spinal column the disease may be situated.

If this should be in the cervical region, the instrument requires the addition of a piece of steel, bent so as to receive the occiput, and attached to the cross-piece by means of a pivot. From either end of this head-piece, which is well padded, a properly-made strap passes under the chin, and thus the head, which would otherwise fall forward until it rested upon the chest, is effectually supported. The pads in this case must, of course, be moved higher up.

If there should be much anterior curvature (incurvation or lordosis) below the point of disease, which may often occur, it is necessary still farther to modify the apparatus, by having but one hinge, and that directly opposite the point of disease. Other changes in the form and bearing of the instrument, which will be obvious to the intelligent practitioner, can easily be made.

Now, let us see how this appliance acts in overcoming the serious difficulties which we meet in the treatment of this disease. The action at the hips and shoulders is evidently directly backward, while at the point of disease it is directly forward. All the points of attachment being unyielding, no force is lost, but it is all exerted in a direction tending to straighten the spine, and to relieve the pressure at the point of disease, which, we have seen, is *the* indication in all these cases.

I have entered at some length into the description of this mode of treatment of this affection, because I have had the opportunity, during the past two years, of comparing it with other methods, and am fully convinced that it is the best that can be adopted. It cannot be said that it is empirical or routine in character, for the apparatus described can easily, and certainly should, be modified to answer the peculiar requirements of every case.

Gymnastic exercises have been recommended by some surgeons in the treatment of this deformity. I will merely say, that experience has proved that they are not only useless but positively harmful, either alone or in conjunction with mechanical treatment, in that form of curvature resulting from spinal *disease.* In lateral curvature, depending upon unequal muscular action, debility, etc., localized exercises may effect the happiest results.

During the progress of Pott's Disease, certain consecutive symptoms are liable to occur, which deserve special mention.

First. Paralysis of the lower extremities, resulting, I believe, excepting in very rare instances, from inflammation extending to the membranes of the spinal cord, and sometimes even to the cord itself. That it is not the result of mechanical pressure exerted upon the cord is evident from the fact, that it is scarcely ever observed in advanced cases after ankylosis has taken place, but in recent cases is quite common, and may be the first premonitory sign of the approach of this disease.

Post-mortem examinations reveal the fact, that however extensive the destruction of vertebræ and the resulting deformity may be, the spinal canal is scarcely encroached upon; the canal and foramina giving exit to the spinal nerves being considerably larger than the size of the organs passing through them require; so that I can see but one cause for the paralysis being permanent—which is *very* rarely the case, though it does sometimes occur—and that is, pressure upon the medulla spinalis by an exostosis. An opportunity of testing the truth of this theory has never occurred. A favorable prognosis may always be given in this form of paralysis. But, though not dangerous, it is liable to recur. Local treatment will scarcely ever be found necessary, perfect rest in bed averting the attack when threatened, or relieving the symptom when present. Paralysis of the upper extremities may occur when the cervical vertebræ are affected.

Second. Malformation of the thorax by the elevation of the ribs and sternum, thereby increasing the width but diminishing the length of that cavity. The intelligent mind will recognize in this change a wise provision of nature, to allow space in which the heart and lungs may perform their respective functions, unimpeded by any mechanical restraint. This apparent deformity will disappear as the posterior curvature is relieved.

Third. Cold abscesses. These may show themselves in the lumbar, iliac, or inguinal regions. When occurring in the last-named situation, they are preceded or accompanied by contractions of the psoas and iliacus internus muscles, and if these contractions are promptly relieved—which may be done, in all cases, by increasing the sustaining force of any apparatus worn by the patient—their formation may be prevented, and the pursuance of the same course will, in some cases, cause the absorption of the matter when abscess has already formed. In whatever situation these abscesses appear, if their absorption cannot be promoted, they should be properly opened at once. If allowed to open themselves, or if the operation be improperly performed, an extensive secreting surface is laid open which will discharge for a long time, seriously impeding the progress of the patient towards recovery. To avoid this result, the abscess should be opened early, and not in its most dependent part, but at the top of the tumor. I mean its top in relation to the axis of the trunk. The operation should be performed with a small trochar, and after the pus is discharged, the tube should be cautiously withdrawn, care being taken to prevent the introduction of air, and a small compress placed over the opening, and fastened down firmly by strips of adhesive plaster applied over the surface in various directions. If the sac thus evacuated fills again, as it sometimes does, it will not be so large as formerly, and the same process must be repeated until its final disappearance.

Any general constitutional disturbances which may occur during the progress of the disease, must be treated as in other cases.

